# Association of Total Calcium With Serum Uric Acid Levels Among United States Adolescents Aged 12–19 Years: A Cross-Sectional Study

**DOI:** 10.3389/fmed.2022.915371

**Published:** 2022-06-10

**Authors:** Fang Gu, Xiaoming Luo, Xiaoli Jin, Changshou Cai, Wenyan Zhao

**Affiliations:** ^1^Center for Reproductive Medicine, Department of Pediatrics, Zhejiang Provincial People’s Hospital, Affiliated People’s Hospital, Hangzhou Medical College, Hangzhou, China; ^2^Department of Neurosurgery, The Central Hospital of Haining, Haining, China; ^3^Center for General Practice Medicine, Department of General Practice Medicine, Zhejiang Provincial People’s Hospital, Affiliated People’s Hospital, Hangzhou Medical College, Hangzhou, China

**Keywords:** total calcium, serum uric acid, adolescents, NHANES, metabolism

## Abstract

**Aims:**

Evidence of a link between total calcium (CA) and serum uric acid (SUA) is absent in adolescents. Thus, this study aimed to determine the relationship between total CA levels and SUA levels in United States adolescents.

**Methods:**

A cross-sectional study analyzed a sample composed of 8,309 United States adolescents aged 12–19 years from the National Health and Nutrition Examination Survey database (1999–2006 survey cycle). Multivariable linear regression analyses and multivariable logistic regression analyses were used to assess the correlation of total CA with SUA levels and hyperuricemia. Furthermore, the dose-response relationship of total CA and SUA levels was analyzed using smooth curve fitting (penalized spline method).

**Results:**

According to multivariable linear regression analysis, every 1 mg/dL increase in total CA level is associated with a 0.33 mg/dL (β = 0.33, 95% CI: 0.27–0.40, *p* < 0.001) increase in SUA. Multivariable logistic regression analyses showed that every 0.1 mg/dL increase in total CA level is linked with an 8% increased risk of hyperuricemia (OR = 1.08, 95% CI: 1.06–1.10, *p* < 0.001). The analyses of smooth curve fitting revealed that total CA levels were linearly correlated with SUA levels (*P*_*non–linearity*_ = 0.152). The results were highly stable in all subgroups. The interaction analysis results presented that race/ethnicity had an interactive role in associating total CA with SUA levels.

**Conclusions:**

In United States adolescents, total CA levels were linearly and positively correlated with SUA levels.

## Introduction

It is well known that serum uric acid (SUA) is a biological product of purine metabolism. SUA is a useful antioxidant in the human blood, and helps maintain antioxidant stress (1). However, elevated SUA can lead to a series of diseases, such as hyperuricemia, gout, hypertension, obesity, diabetes, coronary disease, and chronic kidney disease (2–7). In the United States, elevated SUA is more and more prevalent among children and adolescents (8). In addition, elevated SUA can affect a child’s physical and mental health. Thus, elevated SUA or hyperuricemia has attracted extensive attention from relevant researchers worldwide. However, the pathophysiology of elevated SUA is not fully understood.

Calcium (CA) plays a vital role in several biological processes, such as hormone regulation, blood clotting, muscle contraction, nerve transmission, blood pressure regulation, and enzyme activation (9, 10). Uric acid and CA acetate are the two main elements in urine that contribute to the development of urinary stones, except for oxalate (11). However, the relationship between total CA and SUA remains unclear.

Observational studies have suggested that total CA levels are associated with SUA in adults (12–14), lacking evidence and remaining unclear (12, 13). Another cross-sectional study from Asia also showed a link between total CA levels and the prevalence of hyperuricemia in adults over 40 years (15). However, there are few studies on the relationship between total CA, SUA, and hyperuricemia in adolescents. A better grasp of the link between total CA, SUA, and hyperuricemia in adolescents is needed as it can provide more information for disease surveillance and clarify specific mechanisms.

The present analysis collected data from the National Health and Nutrition Examination Survey (NHANES) from 1999 to 2006. This research examined the relationship of total CA with SUA in adolescents aged 12–19 years.

## Materials and Methods

### Study Design and Population

As part of this study, the data we used were pooled from four 2-year cycles of the NHANES (1999–2006), which is a nationwide cross-sectional survey among non-institutionalized citizens aged ≥2 months old in the United States (16, 17). The National Center for Health Statistics (NCHS) conducted these cross-sectional surveys. The NCHS Ethics Review Board approved the conduct of NHANES. Participants 18 years of age or older provided written informed consent themselves. Written informed consents were provided by guardians or parents for participants aged <18 years. Please log on to www.cdc.gov/nchs/nhanes/ to obtain more details about NHANES.

### Study Variables

In this study, the exposure variable was total CA, and the outcome variable was SUA. Between 1999 and 2001, they were both measured using Roche-Hitachi Model 917 Multichannel analyzers (Roche Diagnostics, Indianapolis, IN, United States). A Beckman Synchron LX20 (Beckman Coulter, Inc., Brea, CA, United States) was used in 2002. We compared the distributions of total CA results from the two laboratories at the transition time, finding no remarkable differences.

### Other Variables

In addition, the following continuous variables were included: age, mean diastolic blood pressure, body mass index (BMI), mean systolic blood pressure, C-reactive protein (CRP), parathyroid hormone (PTH), vitamin D, phosphorus, glucose, triglycerides, total cholesterol, estimated glomerular filtration rate (eGFR, mL/min per 1.73 m^2^), and the use of CA (mg) supplementation. Categorical variables included: sex, race/ethnicity, education, comorbidities (diabetes, hypertension), and physical activity. For more information on how total CA, SUA, and other variables were measured, please visit www.cdc.gov/nchs/nhanes/. eGFR was calculated *via* the creatinine based on the following formula: eGFR = 141 × min (Scr/κ, 1)^α^ × max (Scr/κ, 1)^–1^.^209^ × 0.993*^Age^* × 1.018 [if female] × 1.159 [if black], where Scr is serum creatinine, κ is 0.7 for females and 0.9 for males, α is −0.329 for females and −0.411 for males, min indicates the minimum of Scr/κ or 1, and max indicates the maximum of Scr/κ or 1 (18, 19).

### Statistical Analyses

All estimates were calculated based on NHANES sample weights as recommended by the CDC guidelines. Proportions ± standard error (SE) was used to describe categorical variables. Means ± SE was used to describe continuous variables. This study analyzed participants’ general characteristics in different groups using weighted chi-square tests and linear regression models. Multivariable linear regression analyses were applied to estimate the correlation between total CA and SUA levels. We showed three models simultaneously: the unadjusted model, no adjusted; the minimally adjusted model, we adjusted for sex, race/ethnicity, age; and the multivariable-adjusted model, we adjusted for the covariates in the minimally adjusted model and other covariates. Whether or not the covariates were adjusted was based on the following criteria: whether the covariates altered the regression coefficient by at least 10% when added to the model.

Further, we performed a sensitivity analysis to test the robustness of the results. First, the total CA was converted into a categorical variable of tertile. The P for trend was calculated. We aimed to examine whether the results were consistent with those of total CA as a continuous variable. Second, we analyzed the relationship between total CA and hyperuricemia using multivariable logistic regression analysis. For adolescents, the definition of hyperuricemia is controversial. Some studies suggested that the risk of hypertension would increase when an SUA was ≥5.5 mg/dL (8, 20). Therefore, in our analysis, hyperuricemia or elevated SUA levels were defined when an SUA was ≥5.5 mg/dL. Third, stratified analyses and interactions were implemented according to age, sex, race/ethnicity, education, eGFR, PTH, and phosphorus levels.

We used smooth curve fittings (penalized spline method) to evaluate the dose-response relationship between total CA and SUA levels.

All tests were two-sided and statistical significance was set at *p* < 0.05. All analyses were performed with EmpowerStats^1^, (X&Y Solution, Inc., Boston, MA, United States) and Free Statistics software versions 1.5 2using the R statistical software package^2^, (The R Foundation for Statistical Computing, Vienna, Austria).

## Results

### Participant Selection

**FIGURE 1:**
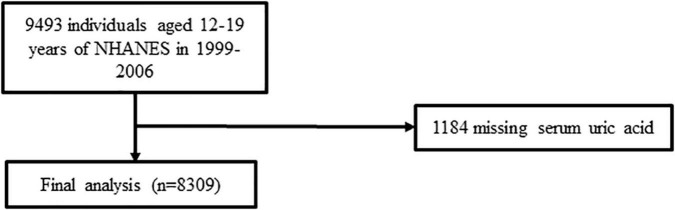
Flow chart of participants.

The flow chart for this study’s participant choice can be viewed in Figure 1. A total of 8,309 eligible adolescents were enrolled after excluding participants with missing SUA values (*n* = 1,184).

### Baseline Characteristics

Table 1 shows the weighted characteristics of the selective participants according to the total CA tertile. The ranges of total CA for tertile 1–3 were <9.4, 9.4 –9.7, and >9.7 mg/dL, respectively. Of these participants, the average age was 15.5 years, and 51.3% were male. Compared to participants in the T1 and T2 groups, those in the T3 group had a higher level of physical activity, increased vitamin D, phosphorus, total cholesterol, glucose, SUA, and had a lower BMI, PTH, and CRP.

**TABLE 1:** Characteristics of study participants.

Characteristics	Total calcium, mg/dL				*P*-value
	Total	Tertile 1	Tertile 2	Tertile 3	
		(<9.4)	(9.4–9.7)	(>9.7)	
*N* *	8,309	2,064	2,850	3,395	
Age, years	15.5 (0.1)	16.1 (0.1)	15.5 (0.1)	15.1 (0.1)	< 0.001
Male,%	51.3 (0.9)	33.4 (2.1)	50.9 (2.2)	62.9 (1.6)	< 0.001
Race/ethnicity,%					
Non-Hispanic white	59.4 (2.1)	55.5 (3.4)	59.1 (2.4)	62.1 (2.7)	0.111
Non-Hispanic black	14.3 (1.7)	13.3 (2.2)	13.8 (1.9)	15.2 (2)	0.393
Mexican American	11.1 (1.4)	14.3 (2.1)	12.7 (1.5)	7.8 (1.2)	0.000
Other Hispanic	7.8 (1.5)	6.2 (1.2)	6.7 (1.4)	9.5 (2.6)	0.123
Other race/ethnicity	7.5 (1.4)	10.7 (2.3)	7.7 (1.4)	5.3 (1.5)	0.020
Education,%					
Less than high school	83.1 (0.9)	78.1 (1.9)	83 (1.7)	86.4 (1.1)	< 0.001
High school	8.6 (0.6)	10.2 (1.3)	9.9 (1.2)	6.5 (0.7)	0.000
More than high school	8.3 (0.7)	11.8 (1.6)	7 (0.9)	7.1 (1)	0.025
Comorbidities,%					
Diabetes	0.6 (0.1)	0.5 (0.2)	0.9 (0.4)	0.4 (0.2)	0.473
Hypertension	3.9 (0.6)	4.2 (1.3)	4.2 (1)	3.4 (0.9)	0.617
Physical activity,%					
Sedentary	15.7 (1.7)	17.7 (2.5)	15.7 (1.8)	13.8 (2.9)	0.277
Low	22.9 (1.3)	26 (3.1)	21.7 (2.4)	21.2 (1.9)	0.209
Moderate	15.4 (0.9)	15.2 (1.7)	16.4 (1.7)	14.6 (1.8)	0.835
High	46 (1.6)	41.2 (2.8)	46.2 (3.1)	50.4 (3)	0.037
Physical examination					
BMI, kg/m^2^	23.3 (0.1)	24.3 (0.2)	23.2 (0.2)	22.7 (0.2)	< 0.001
Mean systolic, mmHg	109.2 (0.3)	109.3 (0.5)	109.1 (0.4)	109.2 (0.4)	0.926
Mean diastolic, mmHg	62.7 (0.4)	62.5 (0.6)	62.5 (0.5)	63.1 (0.5)	0.296
Laboratory data					
Parathyroid Hormone, pg/mL	42.2 (24.1)	46.1 (31.3)	43 (22.2)	39.9 (21.5)	< 0.001
Vitamin D, ng/mL	23.9 (0.4)	22.6 (0.8)	23.5 (0.5)	25 (0.5)	0.013
Phosphorus, mg/dL	4.2 (0.0)	4.1 (0.0)	4.2 (0.0)	4.4 (0.0)	< 0.001
Total Cholesterol, mg/dL	163.2 (0.9)	159.1 (1.3)	162.2 (2)	166.6 (1.3)	0.000
Triglycerides, mg/dL	94.2 (0.0)	92.1 (1.9)	93.5 (2.7)	94.5 (2.8)	0.535
Glucose, mg/dL	91.9 (0.4)	90.5 (0.8)	91.6 (0.7)	93.3 (0.7)	0.027
CRP, mg/dL	0.2 (0.0)	0.2 (0.0)	0.2 (0.0)	0.1 (0.0)	0.003
eGFR, ml/min per 1.73 m^2^	139.1 (0.9)	138 (0.9)	139.1 (1)	139.9 (1.5)	0.240
Serum uric acid, mg/dL	5.1 (0.0)	4.8 (0.1)	5.1 (0.0)	5.3 (0.1)	< 0.001
Supplement use					
Calcium, mg	25.2 (3.2)	20.6 (4.0)	32.7 (6.1)	22.3 (4.5)	0.986

Note: Continuous variables were presented as mean (SE), calculated by weighted linear regression model. Categorical variables were presented as proportions (SE), and calculated by weighted chi-square test. *Unweighted number of observations in dataset. Abbreviation: CRP, C-reactive protein; eGFR, estimated glomerular filtration rate.

### Association Between Total Calcium and Serum Uric Acid

Table 2 shows the link between total CA and SUA. In the non-adjusted model, the total CA level was positively linked with the SUA level. A positive association between total CA and SUA was observed in the multivariable-adjusted model. Every 1 mg/dL increase in total CA level was linked with a 0.33 mg/dL increase in SUA (β = 0.33, 95% CI: 0.27–0.40, *p* < 0.001). At the same time, total CA was converted to a categorical variable (tertile). There were no changes in the trends, and *P* values in all of the models were <0.001.

**TABLE 2:** Association of total calcium with serum uric acid.

Calcium, mg/dL	SUA, mg/dL, β (95% CI), *p*-value		
	Non-adjusted model	Minimally adjusted model	Multivariable adjusted model
Per 1-mg/dL increment	0.64 (0.56, 0.71) < 0.001	0.27 (0.20, 0.33) < 0.001	0.33 (0.27, 0.40) < 0.001
Tertile			
T1 (<9.4)	Ref	Ref	Ref
T2 (9.4–9.7)	0.24 (0.17, 0.31) < 0.001	0.09 (0.03, 0.15) 0.003	0.12 (0.06, 0.17) < 0.001
T3 (>9.7)	0.51 (0.44, 0.58) < 0.001	0.20 (0.14, 0.26) < 0.001	0.24 (0.19, 0.30) < 0.001
*P* for trend	<0.001	<0.001	<0.001

*Non-adjusted model:no covariates were adjusted. Minimally adjusted model:adjusted for age, sex, race/ethnicity.Multivariable adjusted model: adjusted for age, sex, race/ethnicity, education status, physical activity, body mass index, mean systolic, mean diastolic, parathyroid hormone, Vitamin D, phosphorus, triglycerides, glucose, C-reactive protein, and estimated glomerular filtration rate. Abbreviation: SUA, serum uric acid; 95% CI, 95% confidence interval.*

### Association Between Total Calcium and Hyperuricemia

Table 3 showed that an elevated total CA was associated with an increased risk of hyperuricemia. In the multivariable-adjusted model, every 0.1 mg/dL increase in total CA level was linked with an 8% increased risk of hyperuricemia (OR = 1.08, 95% CI: 1.06–1.10, *p* < 0.001). Compared to participants with a total CA level lower than 9.4 mg/dL, the risk of hyperuricemia increased by 30% in those with a total CA level of 9.4–9.7 mg/dL. In addition, there was a 76% increased risk of hyperuricemia in those with a total CA level greater than 9.7 mg/dL.

**TABLE 3:** Association of total calcium level with hyperuricemia.

Calcium, mg/dL	Hyperuricemia, OR (95% CI), *p*-value		
	Non-adjusted model	Minimally adjusted model	Multivariable adjusted model
Per 0.1-mg/dL increase increment	1.1 (1.09,1.12) < 0.001	1.04 (1.03,1.06) < 0.001	1.08 (1.06,1.10) < 0.001
Tertile			
T1 (<9.4)	Ref	Ref	Ref
T2 (9.4–9.7)	1.45 (1.28,1.65) < 0.001	1.14 (0.98, 1.32) 0.081	1.30 (1.10,1.54) < 0.002
T3 (>9.7)	2.19 (1.93,2.47) < 0.001	1.39 (1.20, 1.60) < 0.001	1.76 (1.49,2.08) < 0.001
*P* for trend	<0.001	<0.001	<0.001

*Non-adjusted model:no covariates were adjusted.Minimally adjusted model:adjusted for age, sex, race/ethnicity. Multivariable adjusted model: adjusted for age, sex, race/ethnicity, education status, physical activity, BMI, mean systolic, mean diastolic, parathyroid hormone, Vitamin D, phosphorus, triglycerides, glucose, C-reactive protein, and estimated glomerular filtration rate. Abbreviation: SUA, serum uric acid; 95% CI, 95% confidence interval.*

### The Dose-Response Relationship Between Total Calcium and Serum Uric Acid

**FIGURE 2:**
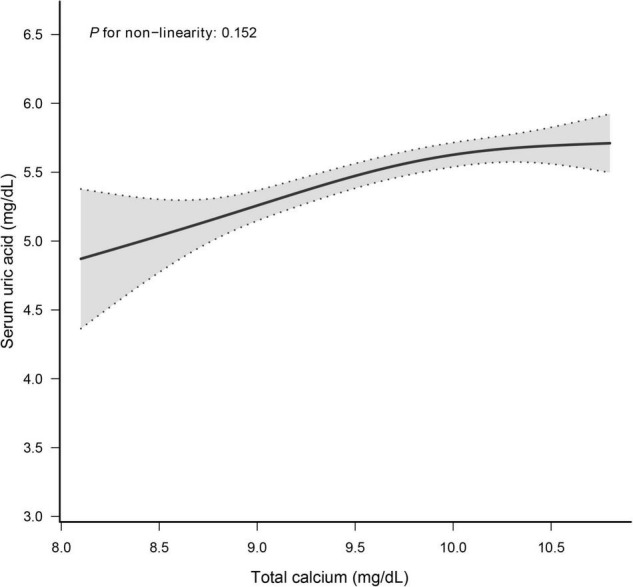
Dose–response relationship between total calcium and serum uric acid. Analyses were adjusted for age, sex, race/ethnicity, education status, physical activity, BMI, mean systolic, mean diastolic, parathyroid hormone, Vitamin D, phosphorus, triglycerides, glucose, C-reactive protein, and estimated glomerular filtration rate.

**FIGURE 3:**
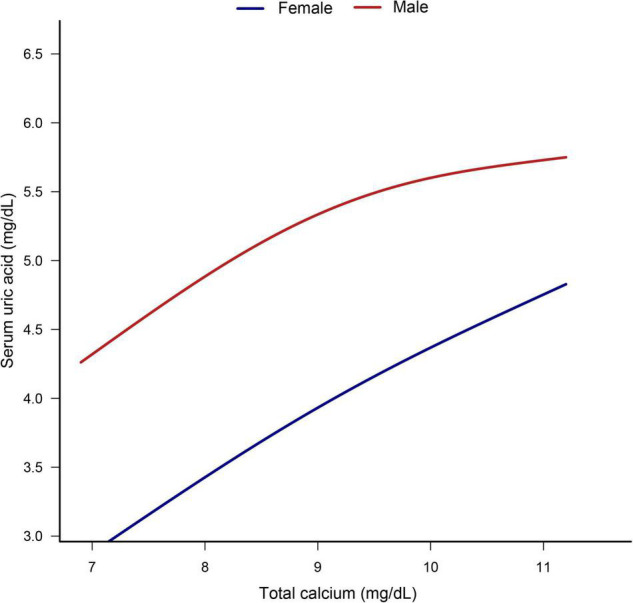
Dose–response relationship between total calcium and serum uric acid stratified by sex. Analyses were adjusted for age, sex, race/ethnicity, education status, physical activity, BMI, mean systolic, mean diastolic, parathyroid hormone, Vitamin D, phosphorus, triglycerides, glucose, C-reactive protein, and estimated glomerular filtration rate.

The smooth curve fitting presented a linear correlation between total CA and SUA levels, with a *P*-value for the non-linearity of 0.152 (Figure 2). We also examined the relationship between total CA and SUA levels stratified by sex. The gender subgroup analysis generated similar findings (Figure 3).

### Sensitivity Analysis

**FIGURE 4:**
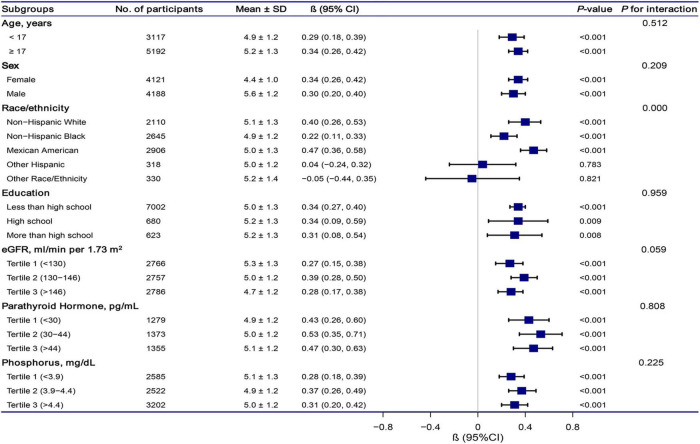
Association between total calcium and serum uric acid according to subgroup. Analyses were adjusted for age, sex, race/ethnicity, education status, physical activity, BMI, mean systolic, mean diastolic, parathyroid hormone, Vitamin D, phosphorus, triglycerides, glucose, C-reactive protein, and estimated glomerular filtration rate. Abbreviations: 95% CI, 95% confidence interval.

The stratification analysis and interaction analysis of the correlation between total CA and SUA are revealed in Figure 4. The results of subgroup analysis were highly consistent with the multivariable linear regression analysis results. The interaction analysis results presented no interactive role in the subgroup except for racial groups.

## Discussion

This cross-sectional study suggested that total CA was linearly and positively associated with SUA in nationally representative US adolescents after adjusting for several major confounding factors. We observed that each 1 mg/dL increase in total CA level was correlated with an increase in SUA of 0.33 mg/dL (β = 0.33, 95% CI: 0.27–0.40, *p* < 0.001). The results were highly consistent in each subgroup. We also found that every 0.1 mg/dL increase in total CA level was correlated with an 8% increased risk of hyperuricemia (OR = 1.08, 95% CI: 1.06–1.10, *p* < 0.001). The analyses of the dose-response relationship revealed total CA and SUA levels were linearly related (Non-linearity = 0.152). According to gender stratification, such an association was effective for girls and boys.

Calcium has been linked to SUA and hyperuricemia in several studies in adults. Data from a 6,337 Chinese population aged 40 years and above found that participants with higher CA concentrations led to a higher prevalence of hyperuricemia (14). Moreover, a cohort study from Ireland suggested that CA levels were higher in people with hyperuricemia when compared with those without hyperuricemia (13). However, not all studies agree with our results. In one Algerian cohort study, patients on hemodialysis showed elevated CA levels linked with lower SUA levels (21). Another descriptive-analytic cross-sectional study suggests that CA levels were not linked with SUA levels in 170 stroke patients (12). However, the sample size was relatively small.

At present, few articles related to total CA and uric acid have been retrieved in PubMed, and the mechanism of the association between total CA and UA is still unclear. We hypothesized that the possible mechanism is as follows: first, both CA and UA are reabsorbed mainly in the proximal tubule of the human kidney. There is a double system responsible for CA extrusion, including the plasma membrane calcium ATPase (PMCA; 22) and the sodium/CA exchanger (NCX1; 23). The expression of PMCA and NCX1 is localized along the kidney’s basolateral membrane, which is involved in CA reabsorption by the kidney, and this procedure is regulated by reactive oxygen species (24). Uric acid is the final product of purine metabolism in humans. Uricase activity has been lost over evolution and renal retention; thus, humans have relatively high UA levels. Uric acid excretion is mainly accomplished by the kidneys. Its reabsorption primary depends on urate transporter 1, a transporter protein located on the surface of renal tubular cells (25). The role of UA in oxidative metabolism is unclear; however, it is known as a powerful antioxidant. It is metabolized in cells to generate superoxide anions and other reactive oxygen species (26). As mentioned, reactive oxygen species contribute to CA reabsorption. Therefore, the association between total CA levels and SUA levels stimulates a new hypothesis about the links between the metabolisms of total CA and SUA. Second, the inflammatory mechanism may explain the relationship between CA and elevated UA. However, CA concentration is positively correlated with inflammation. Some studies showed that hypercalcemia is linked with inflammatory diseases (27, 28). Some crucial inflammatory cytokines, such as IL-6, IL-1β, can upregulate the CA-sensing receptor, which can control blood CA homeostasis and is a promoter and responder of inflammation (24, 29). Meanwhile, IL-6 and TNF-a are essential inflammatory cytokines, positively associated with SUA levels (30–32). Therefore, when UA crystallizes in joints, an elevated level of SUA may cause inflammatory arthritis (33, 34). From the analysis above, we speculate the inflammatory mechanism may influence the positive relationship between total CA and SUA. In general, further studies should be conducted to discover the link mechanism between total CA and SUA.

Subgroup analysis based on gender revealed that for every 1 mg/dL increase in total CA levels, there was a slightly higher increase in SUA in females than in males. It is known that men have higher UA levels than women. Experts suspected that the lower reference value of SUA in women might be related to estrogen inhibiting xanthine oxidase that produces UA (35, 36). However, a large representative cohort study in the US found that gender differences in UA levels are formed during adolescence (37). However, our study found that SUA levels were more likely to be affected by total CA levels in adolescent girls than boys. Thus, novel mechanisms may lead to gender differences in the link between CA and SUA in adolescents. Future studies are needed to verify gender differences in the relationship between total CA and SUA.

In the present study, several strengths are noteworthy. First, this was one of the few studies to directly investigate the positive link between the levels of total CA and the levels of SUA based on a large population of US adolescents. Second, this study used a large sample size, so we performed a subgroup analysis and further found an interaction of race/ethnicity in the association between total CA and SUA levels. Of course, this study also has its limitations. First, this study has an observational character, so it wasn’t easy to permit us to ascertain a causal link between total CA and SUA. Second, our study did not include a history of medication use, such as diuretics, a standard hypertension treatment, which is thought to affect CA and uric acid levels (38, 39).

## Conclusion

The total CA levels were linearly and positively correlated with SUA levels and the risk of hyperuricemia among United States adolescents. Therefore, prospective studies and intervention trials are needed to clarify the mechanism behind the association of total CA and SUA.

## Data Availability Statement

The datasets presented in this study can be found in online repositories. The names of the repository/repositories and accession number(s) can be found below: www.cdc.gov/nchs/nhanes/.

## Ethics Statement

The studies involving human participants were reviewed and approved by the NCHS Ethics Review Board. Written informed consent to participate in this study was provided by the participants’ legal guardian/next of kin.

## Author Contributions

FG: writing—original draft preparation. XL and XJ: validation. CC and WZ: writing—review and editing. All authors contributed to the article and approved the submitted version.

## Conflict of Interest

The authors declare that the research was conducted in the absence of any commercial or financial relationships that could be construed as a potential conflict of interest.

## Publisher’s Note

All claims expressed in this article are solely those of the authors and do not necessarily represent those of their affiliated organizations, or those of the publisher, the editors and the reviewers. Any product that may be evaluated in this article, or claim that may be made by its manufacturer, is not guaranteed or endorsed by the publisher.

## Footnotes

1
http://www.empowerstats.com

2
http://www.R-project.org

## Abbreviations

CA: calcium
SUA: serum uric acid
NHANES: National Health and Nutrition Examination Survey
eGFR: estimated glomerular filtration rate
PTH: parathyroid hormone
CRP: C-reactive protein
NCHS: National Center for Health Statistics
SE: standard error
95% CI: 95% confidence interval.

## References

[1] Wang Q Wen X Kong J. Recent progress on uric acid detection: a review. *Crit Rev Anal Chem.* (2020) 50:359–75. 10.1080/10408347.2019.1637711 31296022

[2] Koto R Nakajima A Horiuchi H Yamanaka H. Serum uric acid control for prevention of gout flare in patients with asymptomatic hyperuricaemia: a retrospective cohort study of health insurance claims and medical check-up data in Japan. *Ann Rheum Dis.* (2021) 80:1483–90. 10.1136/annrheumdis-2021-220439 34158371 PMC8522452

[3] Dalbeth N Choi HK Joosten LAB Khanna PP Matsuo H Perez-Ruiz F Gout. *Nat Rev Dis Primers.* (2019) 5:69. 10.1038/s41572-019-0115-y 31558729

[4] De Becker B Borghi C Burnier M van de Borne P. Uric acid and hypertension: a focused review and practical recommendations. *J Hypertens.* (2019) 37:878–83. 10.1097/HJH.0000000000001980 30620339

[5] Andres-Hernando A Cicerchi C Kuwabara M Orlicky DJ Sanchez-Lozada LG Nakagawa T Umami-induced obesity and metabolic syndrome is mediated by nucleotide degradation and uric acid generation. *Nat Metab.* (2021) 3:1189–201. 10.1038/s42255-021-00454-z 34552272 PMC9987717

[6] Barbieri L Verdoia M Schaffer A Marino P Suryapranata H De Luca G. Impact of sex on uric acid levels and its relationship with the extent of coronary artery disease: a single-centre study. *Atherosclerosis.* (2015) 241:241–8. 10.1016/j.atherosclerosis.2015.03.030 25818387

[7] Srivastava A Kaze AD McMullan CJ Isakova T Waikar SS. Uric acid and the risks of kidney failure and death in individuals with CKD. *Am J Kidney Dis.* (2018) 71:362–70. 10.1053/j.ajkd.2017.08.017 29132945 PMC5828916

[8] Loeffler LF Navas-Acien A Brady TM Miller ER Fadrowski JJ. Uric acid level and elevated blood pressure in US adolescents: national health and nutrition examination survey, 1999-2006. *Hypertension.* (2012) 59:811–7. 10.1161/HYPERTENSIONAHA.111.183244 22353609 PMC3700426

[9] Weaver CM Peacock M. Calcium. *Adv Nutr.* (2019) 10:546–8. 10.1093/advances/nmy086 30915443 PMC6520034

[10] Larsson SC Burgess S Michaëlsson K. Association of genetic variants related to serum calcium levels with coronary artery disease and myocardial infarction. *JAMA.* (2017) 318:371–80. 10.1001/jama.2017.8981 28742912 PMC5817597

[11] Bartges JW Callens AJ. Urolithiasis. *Vet Clin North Am Small Anim Pract.* (2015) 45:747–68. 10.1016/j.cvsm.2015.03.001 26002797

[12] Saadat P Ahmadi Ahangar A Babaei M Kalantar M Bayani MA Barzegar H Relationship of serum uric acid level with demographic features, risk factors, severity, prognosis, serum levels of vitamin D, calcium, and magnesium in stroke. *Stroke Res Treat.* (2018) 2018:6580178. 10.1155/2018/6580178 30057737 PMC6051071

[13] Kumar AUA Browne LD Li X Adeeb F Perez-Ruiz F Fraser AD Temporal trends in hyperuricaemia in the Irish health system from 2006-2014: a cohort study. *PLoS One.* (2018) 13:e0198197. 10.1371/journal.pone.0198197 29852506 PMC5980488

[14] Guessous I Bonny O Paccaud F Mooser V Waeber G Vollenweider P Serum calcium levels are associated with novel cardiometabolic risk factors in the population-based Colaus study. *PLoS One.* (2011) 6:e18865. 10.1371/journal.pone.0018865 21533040 PMC3080882

[15] Liu Z Ding X Wu J He H Wu Z Xie D Dose-response relationship between higher serum calcium level and higher prevalence of hyperuricemia: a cross-sectional study. *Medicine (Baltimore).* (2019) 98:e15611. 10.1097/MD.0000000000015611 31096467 PMC6531036

[16] Curtin LR Mohadjer LK Dohrmann SM Montaquila JM Kruszan-Moran D Mirel LB The national health and nutrition examination survey: sample design, 1999-2006. *Vital Health Stat 2.* (2012) 155:1–39. 22788053

[17] Patel CJ Pho N McDuffie M Easton-Marks J Kothari C Kohane IS A database of human exposomes and phenomes from the US national health and nutrition examination survey. *Sci Data.* (2016) 3:160096. 10.1038/sdata.2016.96 27779619 PMC5079122

[18] Levey AS Stevens LA Schmid CH Zhang YL Castro AF Feldman HI A new equation to estimate glomerular filtration rate. *Ann Intern Med.* (2009) 150:604–12. 19414839 10.7326/0003-4819-150-9-200905050-00006 PMC2763564

[19] Inker LA Schmid CH Tighiouart H Eckfeldt JH Feldman HI Greene T Estimating glomerular filtration rate from serum creatinine and cystatin C. *N Engl J Med.* (2012) 367:20–9. 10.1056/NEJMoa1114248 22762315 PMC4398023

[20] Feig DI Johnson RJ. Hyperuricemia in childhood primary hypertension. *Hypertension.* (2003) 42:247–52. 10.1161/01.HYP.0000085858.66548.59 12900431 PMC1800942

[21] Gouri A Dekaken A Bentorki AA Touaref A Yakhlef A Kouicem N. Serum uric acid level and cardiovascular risks in hemodialysis patients: an Algerian cohort study. *Clin Lab.* (2014) 60:751–8. 24839817 10.7754/clin.lab.2013.130310

[22] Zaidi A Michaelis ML. Effects of reactive oxygen species on brain synaptic plasma membrane Ca(2+)-ATPase. *Free Radic Biol Med.* (1999) 27:810–21. 10.1016/s0891-5849(99)00128-8 10515585

[23] Huschenbett J Zaidi A Michaelis ML. Sensitivity of the synaptic membrane Na+/Ca2+ exchanger and the expressed NCX1 isoform to reactive oxygen species. *Biochim Biophys Acta.* (1998) 1374:34–46. 10.1016/s0005-2736(98)00121-7 9814850

[24] Hoenderop JGJ Nilius B Bindels RJM. Calcium absorption across epithelia. *Physiol Rev.* (2005) 85:373–422. 10.1152/physrev.00003.2004 15618484

[25] Ponticelli C Podestà MA Moroni G. Hyperuricemia as a trigger of immune response in hypertension and chronic kidney disease. *Kidney Int.* (2020) 98:1149–59. 10.1016/j.kint.2020.05.056 32650020

[26] Dawson J Quinn T Walters M. Uric acid reduction: a new paradigm in the management of cardiovascular risk? *Curr Med Chem.* (2007) 14:1879–86. 10.2174/092986707781058797 17627523

[27] Zhang J Sellmeyer DE. Particle disease: a unique cause of hypercalcemia. *Osteoporos Int.* (2020) 31:2481–4. 10.1007/s00198-020-05621-8 32910219

[28] Häusler D Torke S Weber MS. High-dose vitamin D-mediated hypercalcemia as a potential risk factor in central nervous system demyelinating disease. *Front Immunol.* (2020) 11:301. 10.3389/fimmu.2020.00301 32161591 PMC7053380

[29] Anract J Baures M Barry Delongchamps N Capiod T. Microcalcifications, calcium-sensing receptor, and cancer. *Cell Calcium.* (2019) 82:102051. 10.1016/j.ceca.2019.06.005 31276858

[30] Aliena-Valero A Rius-Pérez S Baixauli-Martín J Torregrosa G Chamorro Á Pérez S Uric acid neuroprotection associated to IL-6/STAT3 signaling pathway activation in rat ischemic stroke. *Mol Neurobiol.* (2021) 58:408–23. 10.1007/s12035-020-02115-w 32959172

[31] Ren Q Tao S Guo F Wang B Yang L Ma L Natural flavonol fisetin attenuated hyperuricemic nephropathy via inhibiting IL-6/JAK2/STAT3 and TGF-β/SMAD3 signaling. *Phytomedicine.* (2021) 87:153552. 10.1016/j.phymed.2021.153552 33994251

[32] Zha X Yang B Xia G Wang S. Combination of uric acid and pro-inflammatory cytokines in discriminating patients with gout from healthy controls. *J Inflamm Res.* (2022) 15:1413–20. 10.2147/JIR.S357159 35250292 PMC8896041

[33] Narang RK Dalbeth N. Pathophysiology of gout. *Semin Nephrol.* (2020) 40:550–63. 10.1016/j.semnephrol.2020.12.001 33678310

[34] Dehlin M Jacobsson L Roddy E. Global epidemiology of gout: prevalence, incidence, treatment patterns and risk factors. *Nat Rev Rheumatol.* (2020) 16:380–90. 10.1038/s41584-020-0441-1 32541923

[35] Feig DI Kang D-H Johnson RJ. Uric acid and cardiovascular risk. *N Engl J Med.* (2008) 359:1811–21. 10.1056/NEJMra0800885 18946066 PMC2684330

[36] Huh K Shin US Choi JW Lee SI. Effect of sex hormones on lipid peroxidation in rat liver. *Arch Pharm Res.* (1994) 17:109–14. 10319141 10.1007/BF02974233

[37] Wang Y Charchar FJ. Establishment of sex difference in circulating uric acid is associated with higher testosterone and lower sex hormone-binding globulin in adolescent boys. *Sci Rep.* (2021) 11:17323. 10.1038/s41598-021-96959-4 34462530 PMC8405811

[38] Alexander RT Dimke H. Effect of diuretics on renal tubular transport of calcium and magnesium. *Am J Physiol Renal Physiol.* (2017) 312:F998–1015. 10.1152/ajprenal.00032.2017 28274923

[39] Ben Salem C Slim R Fathallah N Hmouda H. Drug-induced hyperuricaemia and gout. *Rheumatology (Oxford).* (2017) 56:679–88. 10.1093/rheumatology/kew293 27498351

